# Preventative Sensor-Based Remote Monitoring of the Diabetic Foot in Clinical Practice

**DOI:** 10.3390/s23156712

**Published:** 2023-07-27

**Authors:** Evan Minty, Emily Bray, Courtney B. Bachus, Breanne Everett, Karen M. Smith, Emily Matijevich, Maryam Hajizadeh, David G. Armstrong, Brock Liden

**Affiliations:** 1 Cumming School of Medicine, University of Calgary, Calgary, AB T2N 4N1, Canada; 2 Orpyx Medical Technologies, Inc., Calgary, AB T2G 1M8, Canadaemily.matijevich@orpyx.com (E.M.); maryam.hajizadeh@orpyx.com (M.H.); 3 Keck School of Medicine, University of Southern California, Los Angeles, CA 90033, USA; 4 USC Limb Preservation Program, Los Angeles, CA 90033, USA; 5 Southwestern Academic Limb Salvage Alliance (SALSA), Los Angeles, CA 90033, USA; 6 USC Center to Stream Healthcare in Place (C2SHIP), Los Angeles, CA 90033, USA; 7 Cutting Edge Research, Circleville, OH 43113, USA

**Keywords:** diabetic foot ulcers (DFU), remote patient monitoring (RPM), sensor-based monitoring, integrative foot care, chronic disease management, diabetes complications, healthcare costs, sensor-enhanced DFU prevention, activity monitoring, diabetes healthspan

## Abstract

Diabetes and its complications, particularly diabetic foot ulcers (DFUs), pose significant challenges to healthcare systems worldwide. DFUs result in severe consequences such as amputation, increased mortality rates, reduced mobility, and substantial healthcare costs. The majority of DFUs are preventable and treatable through early detection. Sensor-based remote patient monitoring (RPM) has been proposed as a possible solution to overcome limitations, and enhance the effectiveness, of existing foot care best practices. However, there are limited frameworks available on how to approach and act on data collected through sensor-based RPM in DFU prevention. This perspective article offers insights from deploying sensor-based RPM through digital DFU prevention regimens. We summarize the data domains and technical architecture that characterize existing commercially available solutions. We then highlight key elements for effective RPM integration based on these new data domains, including appropriate patient selection and the need for detailed clinical assessments to contextualize sensor data. Guidance on establishing escalation pathways for remotely monitored at-risk patients and the importance of predictive system management is provided. DFU prevention RPM should be integrated into a comprehensive disease management strategy to mitigate foot health concerns, reduce activity-associated risks, and thereby seek to be synergistic with other components of diabetes disease management. This integrated approach has the potential to enhance disease management in diabetes, positively impacting foot health and the healthspan of patients living with diabetes.

## 1. Introduction

Diabetes affects approximately 550 million people (9.3% of the population) worldwide, and the prevalence is projected to increase to 643 million by the year 2030 [1,2]. Of people with diabetes, 34% develop a diabetic foot ulcer (DFU) during their lifetime, half of their DFUs become infected, 20% require hospitalization, 5% lead to lower extremity amputation (LEA), and half of those LEAs lead to LEA of the opposite limb within 5 years [3,4,5,6,7]. Both major and minor amputations have repercussions, such as changes in biomechanics and plantar stiffness, that heighten a patient’s risk of subsequent foot complications [2,8].

The consequences of DFUs are not limited to amputation, and include an increased risk of falls, fractures, reduced mobility, frailty, and mortality [9]. The 5-year mortality rate for those with DFUs is 30.5%, almost identical to the pooled mortality rate of all cancers (31%) [10]. Inevitably, DFUs carry a significant cost to the health system. Annual costs of diabetic foot complications in the U.S. are USD 2.1 billion for emergency department expenses and USD 9.6 billion in hospital admission charges (both amounts adjusted to 2020 USD) [11]. On average, a patient with a DFU has 14 outpatient visits and 1.5 hospitalizations every year, not only driving up healthcare costs but also resulting in lost time from work [11]. More than one-third of patients with DFUs experience symptoms of anxiety or depression, adding to the mental wellness burden on the healthcare system [12].

However, at least 75% of DFUs are preventable using established integrative foot care methods, and DFUs are treatable when detected early [13,14]. Current standard diabetic foot care includes appropriate fitting and/or diabetic footwear, custom insoles (without embedded sensors), education around professional nail care and daily self-checks for redness, callus, and wounds. This care pathway has historically been the same regardless of the patient’s baseline risk profile. Performing a foot self-check can be difficult for patients due to impairments in mobility or vision or lack of recognition of early wound lesions. Adherence to footwear prescription or self-monitoring regimens may also be a challenge for some patients. This can limit the effectiveness of current standard-of-care preventative practice for DFU [15].

Sensor-based remote patient monitoring (RPM) has been proposed as a possible solution to overcome the limitations of the established existing care methods and to establish an integrated healthcare pathway that improves prevention and treatment efficacy for patients with DFUs [16,17,18]. RPM allows healthcare professionals to utilize biometric data from sensor-based devices to detect chronic disease deterioration or exacerbation and enable early intervention to prevent escalation to acute care, providing pathways to care that may be more efficient, more equitable, and tailored to patients’ risk profiles [19,20,21,22,23,24]. The upper bound prevention rate of DFUs may be redefined when sensor-based prevention is paired with effective RPM and foot care best practices.

In diabetes care, RPM has primarily been deployed to support remote review of continuous glucose monitoring data to evaluate insulin dosing, timing, and therapy adherence [24]. RPM has been applied to other chronic disease states, showing early promise in improving outcomes in patients with obstructive pulmonary disease, Parkinson’s disease, hypertension, and other cardiovascular diseases (CVD) [25,26]. The American Heart Association published a position statement providing guidance on RPM implementation to encourage its use to improve CVD outcomes, and systematic reviews examining cardiovascular applications of RPM suggest longer-term cost effectiveness of such approaches [27,28].

There have been limited economic evaluations of sensor-based care and adjunct RPM in DFU prevention. Based on the results of an early pilot study with sensor-based insoles providing direct patient feedback in response to sustained pressure, Markov-based economic modeling suggested that the use of that device to reduce DFU recidivism was cost-effective at device prices of less than USD14,275.50 [29]. Over an 18-month period, expected costs decreased from USD 20,028.69 to USD 5753.19 per patient on average and from USD 54,134.94 to USD 6702.54 per ulcer avoided. The pilot results used to parameterize that model are on the order of those that were subsequently found in a randomized control trial (RCT) evaluating the same device [30] and did not involve notifications directed to the care team as an adjunct to patient-facing alerts. Cost effectiveness has also been suggested in DFU prevention using device-based remote temperature monitoring [31]. In the absence of effect estimates specific to the device used in that study, the model was parameterized using estimates from the literature that involved patient-reported thermometry results to a study nurse [32] and suggested cost savings of USD 8027 per patient. There is a need for studies that evaluate the combined impact of patient-facing and care team notification-based RPM, that include impacts of false positive notifications in cost models.

This perspective article offers insights from deploying sensor-based RPM through digital DFU prevention regimens. First, we summarize the data domains and technical architecture that characterize existing commercially available solutions. We then highlight key elements for effective RPM integration based on data from these domains, including appropriate patient selection and robust background data collection to contextualize biometric data from patients outfitted with sensory technology. The importance of data collection across multiple physical domains of monitoring is emphasized, and guidance on how RPM systems can be effectively integrated into a system of care is offered. This includes considerations with respect to predictive system management and guidance on establishing escalation pathways for remotely monitored patients for both the RPM service provider and the prescribing clinician. Finally, it is suggested that the impact of using sensory technology in monitoring the diabetic foot should be synergistic with other disease management principles in diabetes and to the care of the patient as an individual. By seeking to reduce foot health concerns associated with activity, sensor-based monitoring of the diabetic foot should look to enable the cardiovascular and metabolic risk reduction that can result from gradual activity increases, thereby aiming to improve both foot health and the healthspan of patients living with diabetes.

This perspective is foundationally based on established standards of care, such as those developed by the International Working Group on the Diabetic Foot (IWDGF) and Prevention of Amputation in Veterans Everywhere (PAVE) [33,34]. It has been expanded based on existing sensor-based DFU prevention research and the experiences gained by the authorship through the deployment of RPM through sensor-based digital therapeutic devices. There are limited frameworks and no guidelines available on how to incorporate or respond to data collected through sensor-based RPM in DFU prevention. This perspective addresses this knowledge gap and offers a suggested framework on how to incorporate sensor-based remote patient monitoring, in the context of the diabetic foot, into clinical and preventative care regimens. Collectively, these sources offer insights to other researchers and clinicians looking to sensor-based RPM care as a pathway to save limbs and lives.

## 2. Technology Architecture and Implementation

The following section provides an overview of the sensor technology architecture and implementation of three sensor-based remote RPM products aimed at preventing DFUs. To our knowledge, there are three commercially available products in this space, and these include the Podimetrics SmartMat™ (Podimetrics, Inc., Somerville, MA, USA), Siren Socks (Siren Care, San Francisco, CA, USA), and the Orpyx SI^®^ Sensory Insole System (Orpyx Medical Technologies Inc., Calgary, AB, Canada). Temperature monitoring is a common functionality among all devices. The product form factor dictates capacities for once a day or more frequent measurements in the temperature domain, and the products vary in their thermistor array implementation and sampling frequency. Pressure and activity monitoring are implemented in one product, and we explore the technical architecture and data feedback mechanisms available for data in those domains. We summarize a comparison between these products in Table 1.

### 2.1. Temperature Monitoring

All commercially available devices fundamentally monitor temperature differentials on the plantar surface of the foot and note when the temperature at corresponding locations persistently exceeds an “asymmetry threshold”, indicating potential inflammation of the warmer region. These comparisons are most typically evaluated at corresponding high-risk contralateral locations [35,36,37,38] but more recently have explored ipsilateral temperatures as comparators [39]. The form factor, use case, and data processing logic varies from product to product.

*Orpyx SI^®^ Sensory Insole system*: The Orpyx SI^®^ Sensory Insole system contains temperature sensors embedded into an insole that are then placed into the user’s footwear for use during their daily activities. After an acclimation period, the sensors track temperature on the plantar surface of the foot at a frequency of one measurement per minute. The assembled device has been tested over a range of 15–40 °C via immersion in a thermostatic water bath and demonstrated an accuracy of ±0.6 °C when compared to a reference standard (510(k) submission ID K231880, pending public release). The sensor data are stored on board and wirelessly transmitted via Bluetooth to a HIPAA-compliant server to be processed and stored. The daily average difference is calculated from all measurements during that day, and comparisons are made both contralaterally (against the sensor location on the contralateral side) and ipsilaterally. Analogous to previous RCTs examining temperature monitoring in DFU detection, when two consecutive daily average measurements exceed a 2.2 °C difference threshold [32,40,41], RPM staff are notified via a flag in a HIPPA-compliant dashboard. At the time of this manuscript, temperature differentials are reported for the 1st, 3rd, and 5th metatarsals and the heel. A hallux temperature sensor is embedded in the insole and is the subject of current research and development prior to its public release.

*Siren Socks:* The Siren Socks are comprised of six temperature sensors woven directly into the sock fabric to continuously measure temperature across the plantar surface of the foot. The sensors track temperature at 10 s intervals across six areas of the foot: the hallux, metatarsal points 1, 3, and 5, the midfoot, and the heel. The stand-alone sensors have been tested over a range of 20–40 °C via immersion in a thermostatic water bath and demonstrated an accuracy of ±0.2 °C when compared to a reference standard [37]. The sensors embedded in socks were similarly evaluated in a thermostatic water bath and were reported to show high agreement with the reference standard [37]. The sensors connect to a small tag on the sock that houses a microcontroller, battery, and Bluetooth chip, which stores temperature data [37]. Data are transmitted to the cloud through a wireless cellular data hub that connects to the Bluetooth chip on the sock. Data are transmitted both to the physician-facing web portal and patient-facing mobile device. Temperature asymmetry is evaluated at six contralaterally matched locations, and the daily average differential is computed. Temperature asymmetries that exceed a threshold trigger a warning to the clinical staff [31,37,38,42].

*Podimetrics SmartMat*™: The SmartMat™ is an at-home once-daily use wireless floor mat that contains a high-density array of approximately 1000 thermistor sensors [43]. The patient is instructed to stand on the mat and remain stationary for 20 s while the device records a thermogram of both feet. The thermogram is reported to have an accuracy of ±0.6 °C and a precision of 0.1 °C, and the device is accurate over a range of 15–40 °C [35]. Once the scan is complete, data are de-identified and securely and wirelessly transmitted to a HIPAA-compliant server to be processed and stored. The daily left versus right temperature asymmetry is automatically calculated based on the thermogram and compared to an asymmetry threshold. Temperature asymmetry is evaluated at six contralaterally matched locations: the hallux, first, third, and fifth metatarsal heads, midfoot, and heel. When patients are missing part of the foot due to amputation, temperature measurements from a nearby location on the foot are used. Temperature asymmetries that exceed a threshold trigger a warning to the clinical staff [35,36,44].

### 2.2. Pressure Monitoring

Pressure overload plays a central role in models of DFU pathogenesis, with different accounts as to the role of peak pressures, shear pressure, or the impact of exceeding capillary perfusion pressure across a pressure time integral [40,45,46]. It is common practice to prescribe custom footwear or insoles to offload areas of presumed high plantar pressure [47]. A complementary approach, which has shown promise in randomized controlled trial evaluation, has been the provision of patient-directed feedback with regard to the areas of the foot experiencing sustained pressure [30]. This approach can complement the provision of a custom insole prescription. Of the three commercially available sensor-based DFU RPM systems, only one has architecture to support measurement in the pressure domain.

The Orpyx SI® Sensory Insole system contains 22–37 force-sensitive resistors (FSRs, exact number depends on the insole size) embedded in each insole. The design reflects a pressure monitoring regimen designed to detect pressures that, when sustained over time, can cause tissue ischemia. FSRs are calibrated at manufacturing time to a tolerance between 35 and 50 mmHg and operate as a switch at that threshold. This value was chosen based on estimates of capillary perfusion pressure at the foot [48]. Over a 15 min sliding window, if 95% of sensor pressure readings exceed the threshold, the sensor is marked as being in a “high-pressure state” [30]. Each insole is divided into six anatomical regions to simplify pressure data interpretation, namely a heel, midfoot, medial metatarsal, lateral metatarsal, medial toe, and lateral toe region. If any sensors in a region are in a high-pressure state, that region is considered to be in a high-pressure state.

In the Orpyx SI® system, pressure data have two paths for feedback: patient-facing and clinician-facing. For the clinician-facing feedback, when any combination of regions is in a high-pressure state for greater than 40% of usage time for a day, a warning highlighting a pressure data trend of concern is generated for RPM review. For the patient-facing feedback, when a sensor region is in a high-pressure state, the patient is provided with real-time cues for pressure offloading through an app-based display. Patient-facing biofeedback is continuous, but patients are cued no more than once per hour to balance user engagement and alert fatigue [49].

### 2.3. Activity Monitoring

There are no claims evident in the literature that describe the activity monitoring capabilities of two of the three commercially available systems. The Orpyx SI sensory insole system contains an inertial measurement unit (IMU) embedded in the sensory insole to record foot motion. A step count algorithm is used to report daily step counts based on signals from a triaxial accelerometer. Step count data are wirelessly and securely uploaded for display in the HIPAA-compliant dashboard.

## 3. Patient Selection

Patient selection is a very important component of any remote monitoring program to ensure that it is effective for both the patient and the treating clinician.

Based on factors that deem a patient to be at risk of developing a DFU, and the importance of engagement in the success of RPM programs, it is recommended that patients have the following characteristics [33]:

- Patients with Type 1 or Type 2 Diabetes with established peripheral neuropathy (PN) and loss of protective sensation (LOPS) as established by the Semmes–Weinstein monofilament test;
- Patients with a previously healed DFU (no active wound present). This is referred to as a person in remission from a diabetic foot ulcer [3,50,51];
- Patients who are willing and open to engaging in their diabetic foot health through digital prevention and an RPM service;
- Patients with the cognitive capacity and technological fluency to understand the digital device and its operation;
- A supportive care environment is also an asset but does not preclude the possibility of benefit from RPM.

These patient selection criteria generally align with risk levels established by international clinical practice guidelines such as PAVE and those written by the IWGDF [33,34]. The alignment of sensor-based care and RPM with the risk levels established by these organizations is outlined in Table 2.

The performance of some monitoring domains may be adversely affected by a patient’s comorbidity profile. When selecting the specific physiologic data collected by an RPM solution, it is important to consider any chronic condition(s) that the patient may have, which may influence data trends. Patients may exhibit chronic limb temperature differences as a variant of normal physiology or mediated by asymmetries in peripheral arterial disease (PAD) or other structural or neurological compromise. All of these may predispose to false negatives or false positives in the temperature domain [35,52,53,54]. Temperature monitoring may also be impacted by patient immunocompromise, which could serve to partially suppress an inflammatory response to tissue injury. This, as well as immunosuppressive impacts of other commonly occurring comorbidities in people with diabetes (such as end-stage renal disease (ESRD) in the generation of temperature differentials in the diabetic foot, remain understudied [55].

RPM programs should be designed to include comorbid patients, not exclude them, as these patients are often at high-risk for DFU development. By collecting data across multiple modalities at different points of DFU risk and pathogenesis pathways and the provision of a service that emphasizes best practices in self-care, we feel that such patients can benefit from digitally enhanced risk reduction through RPM.

## 4. Clinical Assessment and Data Collection

Prior to enrollment in a remote monitoring program, a diabetic foot and wound history should be gathered and complemented by a complete medical and surgical history, social history, nutritional history, and physical exam as it pertains to wound healing potential in the lower extremities. This helps to contextualize the biometric data derived from sensor-based devices and facilitates informed care decisions. Recommended patient history and physical examinations are detailed in Table 3.

A physical examination should be completed that includes vital signs, lower leg assessment (including sensory loss), vascular assessment, gait assessment, documentation of any foot deformities or differences, a detailed wound assessment, and information detailing any environmental factors that affect the biometric measurements.

A patient’s functional status can reveal insights on the localized risk for DFU, as well as broader risks to the patient’s wellbeing. A gait assessment is recommended to focus on the presence of any specific gait abnormalities such as calcaneal gait (high risk to heel region) and compensatory gait patterns such as early knee flexion, foot or hip abduction, or foot drop. These gait patterns can contribute to abnormal forces and lead to potential tissue damage [57]. A functional assessment should also consider the use of any assistive devices (i.e., walker, cane, etc.) or any upstream biomechanical issues that may cause compensation at the foot or ankle (i.e., hip tightness, knee contractures, unilateral weakness, etc.). More broadly, neuropathy places patients at increased fall risk, and a functional assessment should optimally be paired with access to services designed to reduce this risk at home.

In patients with diabetes, there are no clinical signs or symptoms that can accurately exclude PAD [58]. As such, a peripheral vascular disease (PVD) assessment, preferably through an ankle brachial index (ABI) with segmental pressures and waveform analysis (if local expertise exists), should be completed at intervals appropriate to local guidelines [58]. These tests can be helpful in contextualizing temperature data from available digital devices. Some devices and monitoring regimens have excluded patients with PVD in the trials contributing to their evidence base [35,40], while others have included patients with non-limb threatening disease [30,59].

Additional investigations that are recommended to capture a clinician’s records include those relevant to the assessment of diabetic control and end organ damage, as summarized in relevant disease guidelines [60].

## 5. Remote Patient Monitoring for Diabetic Foot Ulcer Prevention: Overview

**Figure 1:**
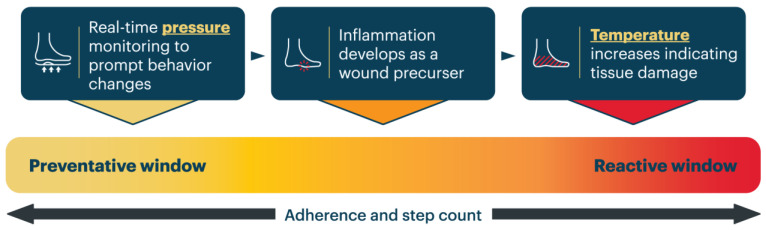
Physiologic parameters contributing to tissue damage on a preventative spectrum. Pressure is the first physiologic parameter that provides insights into formation of a DFU or pre-ulcerative lesion and, thus, is deemed preventative when addressed. Following increased levels of sustained pressure, tissues become inflamed and wound precursors develop. Actions surrounding temperature rooted in DFU or pre-ulcerative lesion formation are considered reactive in nature due to the underlying tissue damage that has taken place and led to the temperature increase being detected.

An RPM team is typically comprised of licensed, qualified healthcare practitioners. This team may be part of the clinical practice or be contracted by a third-party group, often the company that supplies the RPM technology. RPM marries the need for improved continuity of care with that of easing patient accessibility to care, ultimately encouraging a collaborative approach and strengthening the overall healthcare offering. With respect to caring for the diabetic foot, there is a continuum of physiologic parameters that evolves alongside the risk pathway and progression of DFU. Digital prevention, when paired with an RPM service, can be considered preventative of tissue injury or reactive to tissue injury. This important distinction is illustrated in Figure 1.

**Figure 2:**
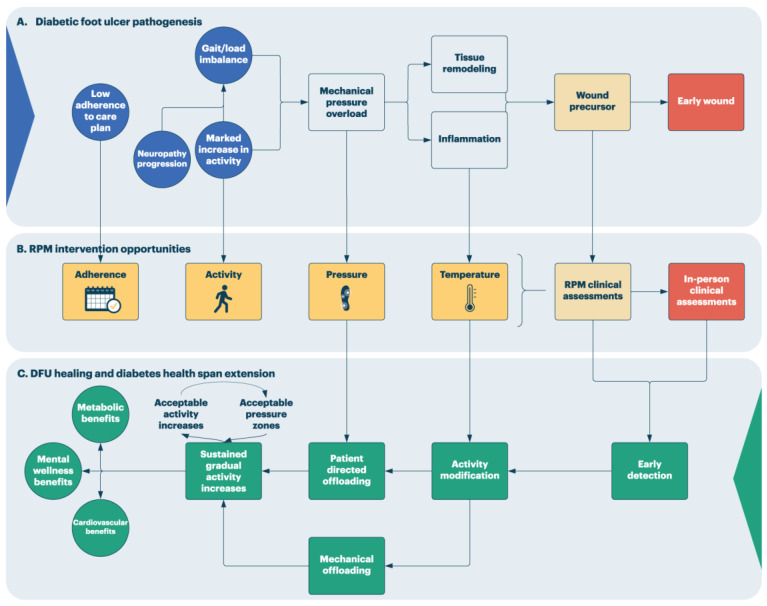
Simplified DFU causal pathway and RPM intervention opportunities.

Figure 2 expands on elements involved in the DFU causal pathway, opportunities for RPM intervention, and, ultimately, maintenance on a path conducive to tissue healing, injury prevention, and diabetes healthspan extension.

The left side of Figure 2A outlines a high-level set of causal factors that contribute to DFU risk. These may relate to disease or comorbidity factors (e.g., neuropathy progression/severity) or have complex social determinants. The latter should contextualize how an RPM service and a clinical team intervene in a case. For example, patients may have to be on their feet due to job or transportation constraints and advising these patients to simply stay off their feet without a highly compelling reason is not a reasonable option. These patients likely benefit from preventative care as far upstream as possible in the DFU pathogenesis pathway. As the pathway progresses, gait/load imbalance, marked increases in activity, or contributions of both lead to a state of pressure overload. There are varying accounts as to the contribution of peak pressures, shear pressure, or the effect of exceeding capillary perfusion pressure across a pressure-time integral to pressure injury [30,47,61]. These states of pressure overload can lead to tissue remodeling and in cases of tissue injury, an inflammatory state [45,62]. These ultimately lead to wound precursor lesions and an early wound.

Panel B of Figure 2 illustrates intervention opportunities for a digital prevention-enabled RPM service. Adherence analysis offers opportunities to promote engagement, not only in digital prevention but also in the broader preventative and therapeutic care plan, in keeping with integrative foot care best practices [46,63]. Pressure analysis can offer opportunities to intervene prior to the development of tissue injury, which can be patient-facing to promote immediate patient-directed offloading and/or RPM-facing to be integrated into RPM assessment decisions [30]. Temperature-based analyses can serve as a sentinel to the development of inflammation and tissue injury, although it suffers from high false positive rates [35,54]. Meanwhile, activity monitoring can add important context to data trends of concern in other domains [64].

The net effect of analytics that highlight concerning data trends is to generate an RPM assessment at a data-driven threshold. If RPM services are provided by an external third party, these assessments represent net new clinical assets to the circle of care instead of depleting existing resources. They can also be accessed directly by the patient, thereby improving their awareness and access to care. RPM assessments play an important role in clinical triage, reducing false positive resource drains on existing clinical care teams by seeking to escalate only the concerning cases for an in-person assessment.

The desired ultimate impact of RPM intervention is to interdict DFU pathogenesis and promote tissue and patient wellbeing, as shown in Figure 2C. Proceeding from right to left, improved access to care and clinical triage via the RPM service aims to provide early detection of pre-ulcerative events. Ideally, interventions occur as far upstream in DFU pathogenesis as possible. Intervening at the inflammatory stage, prior to tissue breakdown, may provide opportunities for activity modification. These may include activity reduction, alternatives to weight-bearing exercise or mechanical offloading as arranged by the clinical team. These offloading strategies may benefit from ongoing monitoring in the pressure domain to ensure the desired offloading or gait retraining effect is achieved. Similarly, offloading interventions can apply when data trends of concern develop in the pressure domain in an attempt to prevent tissue injury altogether. In this domain, patient-facing notifications can serve to empower patients to direct an offloading strategy during their daily activities.

The goal of a DFU prevention RPM system should not only be to prevent DFU, but also to promote sustained, acceptable, and gradual increases in activity and overall wellbeing. Exercise is not contraindicated for those at risk of DFU; however, exercise and physical activity interventions should incorporate gradual, sustained increases in activity to limit foot health adverse events [63,65]. By incorporating techniques in health coaching and motivational interviewing, the RPM team can help direct a case to a desired end state where sustained modest increases in mobility can be monitored for adverse impacts on foot health. When sustained over time, this can lead to the anticipated metabolic, cardiovascular, and mental health benefits of increased exercise [66]. In doing so, digital-based DFU prevention RPM services provide more than just DFU prevention but could potentially provide benefits that impact the diabetes disease trajectory.

## 6. Prediction System Quality Management in Remote Patient Monitoring Systems

*“False positives are one of the worst things you can do to an early warning system” (Chesley Sullenberger, Captain of US Airways Flight 1549, “Miracle on the Hudson”)*

[67]

Sensor-based technologies provide the data inputs for a predictive system that seeks to escalate more concerning cases to clinical attention at a data-driven threshold. Every prediction system needs to balance the risk of false negative and false positive signals. While all seek to minimize the former, the pernicious effects of false positive alerts in clinical environments are well documented in the literature surrounding alert fatigue [68,69]. This is an important consideration when deploying sensor-based technology into a system of care. It is imperative that any RPM system overseeing sensor-based care institutes measures to reduce the introduction of false positive alerts into a busy clinical environment or into the care experience of the patient. To do otherwise risks the necessary engagement with the patient and the treating team, both of which are prerequisites to the success of the care system.

To ensure escalation processes are not adversely impacted by false positives, RPM systems can employ an additional clinical layer into the prediction and assessment process to serve as the first line in alert triage. RPM services that include these additional measures are typically staffed with clinically trained registered nurses (RNs) or other licensed health professionals. This complements existing care teams with additional input to ensure that digital therapeutics do not burden the referring clinical team.

Measures should be instituted to continually evaluate and improve the quality of the prediction system and RPM service being provided. Prediction algorithms benefit from continual efforts in data engineering and ongoing development and evaluation, a field broadly known as machine learning operations [70]. This requires the support of an internal data science team that operates in conjunction with the software and hardware development teams. Additionally, a robust quality system and oversight by trained medical professionals, typically a Doctor of Medicine (MD), Doctor of Osteopathic Medicine (DO), or Doctor of Podiatric Medicine (DPM), further support RPM systems.

## 7. Implementation, Opportunities, and Limitations of Sensor-Based RPM in Clinical Practice

The addition of a sensor-based RPM program to standard clinical practice should be synergistic with patients’ existing care regimens. Successful integration of a sensor-based RPM program should consider the following:

- Patient selection
  ○ Patient selection plays a key role in the success of any RPM program. Patients should be identified and reviewed against patient selection characteristics, such as those outlined in this perspective, prior to patient enrollment in the RPM program. The goals of the program should be discussed with the patient, along with the responsibilities of the patient, clinician, and RPM service provider.
- Escalation and communication
  ○ RPM escalation protocols should be reviewed and agreed upon by the treating clinician and should clearly establish and outline communication methods and response timelines.
  ○ Training of relevant personnel on the sensor-based technology and associated RPM protocols is an important step in the implementation of an RPM program. Training should be provided not only to the patient but also to those involved in the patient’s care, including the treating clinician, clinic staff, and the patient’s support system.
- Technology selection
  ○ The specific hardware and software deployed to collect the remotely monitored data may differ based on patient-specific requirements and needs. In the context of DFU prevention, this may take the form of sensor-embedded wearables such as insoles or socks that can be used throughout daily activities or non-wearable sensor-embedded technology such as a mat or recording device that can be used at home [30,31,35,71].
  ○ The RPM technology should ultimately be selected based on factors that consider the patient’s underlying disease state, lifestyle goals and constraints, technological fluency, and engagement with their overall health. The technology selection will also be influenced by the care providers’ familiarity with the technology and whether the technology is covered by insurance [24].

There is great flexibility in how sensor-based RPM may be deployed to maximize both effectiveness and practicality. Given that sensor-based RPM programs are a relatively new offering, it is important to acknowledge the potential barriers and limitations that exist. These barriers and limitations may include:

- Technological learning curve
  ○ Due to the nature of sensor-based RPM, there is a technological learning curve that patients and clinicians face. Patients that have some experience with technology-based solutions may find it easier to participate in such programs. Patients that are not comfortable with technology or that do not have the appropriate support system to help learn a new technology may face additional barriers to success in a digital-based RPM program and require additional support.
  ○ Other patient factors, such as dexterity and visual impairment, should be acknowledged, depending on the form factor of the technology.
- Patient acceptance and engagement
  ○ Success in a digital, sensor-based RPM program relies heavily on patient engagement with the technology and remote monitoring nurse. It is also helpful for the treating clinician to provide support and encouragement to both the patient and RPM nurse, facilitating a team approach to patient care.
- Data privacy
  ○ It is of the utmost importance that digital, sensor-based RPM providers adhere to standards of digital health information storage. This includes, but is not limited to, the Health Insurance Portability and Accountability Act (HIPAA) in the United States and equivalent provisions in other jurisdictions.

## 8. Patient Assessment through RPM Services

The desired outcome of a well-calibrated prediction of risk in an RPM system is to prompt the involvement of virtual clinical assessment in an RPM process, as depicted in Figure 2. It is suggested that the following general principles apply with respect to remote assessment in an RPM service.

- When data trends of concern are noted, patients are contacted in accordance with clinical guidelines internal to the RPM service provider. This clinical guidance should be re-evaluated at regular intervals and serves to create predictable care processes and reduce unnecessary care variation within the RPM service.
- When patient contact is initiated, it should include an assessment of the physiologic data generating the concerning trend and the clinical context by a licensed healthcare professional.
- Patient contact includes a remote assessment of the patients’ feet (self-guided exam) when possible and reinforces best practices in integrative foot care [13].
- In cases where that assessment reveals visible abnormalities or other signs of clinical concern, the clinician’s office is notified directly.
- In cases where there are no such concerns identified, communication of the data triggering the concern, clinical context, and interaction with the patient proceeds via documentation in the legal record of care shared between the RPM provider and the treating team or through some other reporting mechanism. These cases, as well as true positives, should inform further refinement of the prediction system.

An additional benefit to an RPM service is that patients can be provided with ongoing education and coaching surrounding diet, exercise, medication and integrative foot care [13,72]. Patients immediately forget up to 80% of what their healthcare provider communicated during an office visit, and the value of reinforcement of self-care best practices cannot be overstated [73].

## 9. Clinical Response to RPM Escalation

If a patient presents with any concerning trends in their physiologic data, the remote monitoring healthcare professional may escalate this patient’s information to their treating clinician for review and medical intervention. Clinical escalation parameters are governed by a mutually agreed upon RPM protocol between the clinician and RPM service provider.

**Figure 3:**
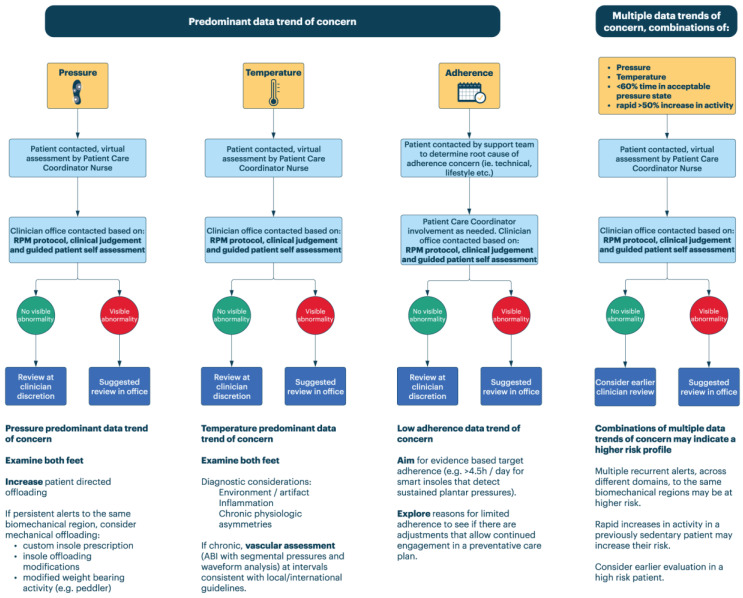
Suggested clinical actions for patients with concerning trends in pressure, temperature, or adherence domains, or across multiple domains.

Figure 3 illustrates the recommended actions for clinicians once a patient has been triaged from remote care to in-office care.

In cases where an in-person clinical assessment ensues, most elements of that interaction (clinical history, physical exam) proceed in ways that remain rooted in the clinician’s existing clinical training and protocols. However, the introduction of digital prevention may introduce additional clinical considerations and opportunities. The possible data trends of concern revealed through RPM digital technologies are elaborated on below. The clinical guidance recommendations provided in this section are suggestions, and clinicians are encouraged to use their judgement on final care decisions.

### 9.1. Pressure Predominant Data Trend of Concern

Pressure monitoring generally provides upstream opportunities in prevention, in theory, prior to the development of inflammation from tissue injury. The following recommendations are provided to support clinicians in their assessment of patients escalated to their attention due to a pressure predominant data trend of concern. Note that these are derived primarily from a pressure monitoring regimen that aims to detect sustained elevated pressures.

- Clinicians may be tempted to focus an exam on a particular region that is generating data trends of concern. While that may indeed be an area of pressure overload, anchoring in a particular region should be avoided. **It is important that both feet are assessed**.
  ○ Early experience suggests pre-ulcerative pathogenesis might see pressure warnings preceding dermal changes in the same region or in different regions.
  ○ This may proceed through known biomechanical mechanisms (e.g., load switching between metatarsal heads 1 and 5), through patient offloading to a contralateral limb, or simply due to a change in activity that causes a broader alert pattern.
- Higher risk situations may involve recurrent data trends of concern to the same biomechanical regions.
  ○ Consider mechanical adjustment if this is persistent.
- Changes in activity levels may generate more cumulative load on the foot tissues and more pressure-related data trends of concern [74]. Some research also suggests that high day-to-day variability in activity, regardless of activity volume, may put individuals at higher risk of ulceration [75]. However, risk in such situations must be balanced against established cardiovascular, metabolic, and mental benefits of increasing mobility in high-risk populations. Digital therapeutics and RPM services need to be aligned with broader goals for healthy living.
- In keeping with those goals, the following clinical actions are suggested:
  ○ In cases of a recent, abrupt increase in activity (absent any evidence as to what is definitively unsafe, a definition of >50% of monthly baseline over a few days is used), consider counselling towards gradual increases instead, if possible [63].
  ○ If patient-facing alerts are provided by the digital prevention device, counsel towards higher interaction so that risks of increases in the activity to the diabetic foot can be more effectively managed through patient offloading [30].
  ○ Through health coaching and techniques in motivational interviewing, and in the absence of pressure data trends of concern or in a setting where they are reliably offloaded, aim for monthly increases in activity of 10% [13].

### 9.2. Temperature Predominant Data Trend of Concern

The following recommendations are provided to support clinicians in their assessment of a temperature-predominant data trend of concern.

- Pre-enrollment vascular assessment (preferably through ABI with segmental pressures and Doppler waveform analysis) at an interval appropriate to local guidelines can be helpful in contextualizing possible perfusion differences [58].
- Clinicians may be tempted to focus an exam on a particular region that is generating temperature data trends of concern. While that may be a focal area of inflammation, anchoring on a particular region should be avoided. **It is important that both feet are assessed**.
- Temperature asymmetry may be driven by areas of relative warmth or coolness.
  ○ Pre-ulcerative inflammation may have a significant lead time, with some studies suggesting >5 days and others suggesting as long as >20 days [32,35]
  ○ Relative temperature differences have low specificity in DFU prediction and may be driven by [54]:
    ▪ Inflammation [37,40,45,54];
    ▪ Environmental factors;
    ▪ Chronic load-bearing differences [76];
    ▪ Perfusion differences (large or small vessel disease asymmetries) [77];
    ▪ Neurological asymmetries (e.g., impaired sympathetic tone) [78];
    ▪ Asymmetries in muscle mass (or other structural asymmetries);
    ▪ Venous insufficiency, edema.
  ○ A meta-analysis of five temperature monitoring RCTs including 772 patients has recently provided low certainty evidence suggesting a risk reduction is associated with home skin temperature monitoring when ambulatory activity is reduced (e.g., by greater than 50% [59]) in response to detected hot spots [16]. Given the prevalence of false positives in the temperature domain [54], the potential impact of such a recommendation on the need for activity promotion in diabetic patients merits some consideration [65]. In view of this, some providers may want to consider other forms of activity modification (e.g., non-weight-bearing exercise). The safety of such approaches should be further studied, and providers should exercise their clinical judgment in balancing foot health concerns against activity promotion goals.

### 9.3. Adherence Predominant Data Trend of Concern

One of the advantages of engaging in digital-based prevention is the insights that can be generated with respect to patient adherence to preventative therapy. Prospective clinical trials with insoles monitoring sustained pressure have demonstrated that in patients with a previous DFU, a threshold of 4.5 h or more of wear per day led to a risk reduction of re-ulceration of 86% [30].

Reasons for limited adherence may be complex. Chronic disease can be exhausting, and patient circumstances can make prioritization of their health difficult. A significant portion, ranging from 40 to 80%, of information conveyed by providers is immediately forgotten, and approximately half the remembered information is incorrect, which may also undermine adherence to a preventative care plan [79]. An additional benefit to an RPM service can be realized by virtue of consistent, recurrent patient engagement with a licensed healthcare provider to provide coaching and overall support. These healthcare providers can work with patients to promote engagement with preventative technology and other preventative and therapeutic care regimens that are compatible with patient preferences and goals.

In keeping with those goals, the following clinical actions are suggested:

- Aim for an evidence-based adherence target (e.g., >4.5 h per day (for insoles that deliver alerts in response to sustained elevated plantar pressure [30]));
- Explore reasons for limited adherence to see if there are adjustments that can be made to allow for continued engagement in a preventative care plan.

Licensed care professionals excel at creating and maintaining therapeutic relationships and at contextualizing care needs to a patient’s preferences. Care remains an intrinsically human endeavor. Those relationships can be a significant asset in chronic disease management, which is something that no digital device alone can completely supplant.

### 9.4. Multiple Data Trends of Concern

Current evidence exists that correlates the increases in pressure time integrals, temperature, and activity, but their independent contributions within a multifactorial casual pathway have not been established [30,35,40,80]. Although speculative, a reasonable assumption is that greater risk may be conferred by multiple data trends of concern across domains of pressure and temperature to the same biomechanical region when occurring in proximity to a concerning activity context. In cases of multiple data trends of concern, clinical action recommendations may include those suggested for individual data trends of concern, as outlined in the above sections. However, earlier escalation should be considered due to the presumed higher risk level when multiple data trends of concern are detected.

## 10. Sensor-Based Preventative Care Will Enhance Our Understanding of DFU Pathogenesis and Promote a Systems-Based Approach to Prevention

*“Everything should be made as simple as possible, but not simpler” (Possibly Albert Einstein, as paraphrased by L. Zukofsky and then R. Sessions)*.

[81]

It is acknowledged that the representation of the DFU pathogenesis pathway in Figure 2 is both simplified and incomplete. Indeed, we expect that the application of sensor-based technology under the insensate diabetic foot at scale will likely lead us to re-evaluate models for DFU pathogenesis. In our search for a causal model for DFU, there is often an assumption that the risk resides in the culprit foot [82]. Collecting large-scale, real-world sensor data from both limbs will highlight the importance of the interplay between both feet through the gait cycle, activity patterns, and other patient factors [83,84,85,86]. Indeed, Petersen and coworkers highlighted that, in remission, recurrence frequently occurs on sites distant from the original lesion, with the leading site for recurrent disease being on the contralateral foot [87]. Current literature recognizes limitations in predicting or detecting ulceration using individual monitoring domains such as temperature thresholds, peak pressure, shear pressure, and total pressure time integrals, or activity [46,47,54,80,86]. This suggests an alternative view, which de-emphasizes the need to define a singular mode of pathogenesis and accepts that when factors interact in a multidetermined system, emergent failures, such as DFUs, are created in ways that are not always directly predictable from the individual components in isolation [88].

This ‘systems’ view of DFU development is complemented by a systems approach to DFU prevention. One component of that prevention system might entrust RCT data in which dynamic patient directed offloading in response to sustained levels of high, but not peak, plantar pressures over a prolonged period in real-world use demonstrated a 71% reduction in DFU recurrence in the intervention group, which increased to 86% in patients adhering to the threshold of 4.5 h/day of use [30,47]. This could be complemented by dermal thermography [16] and activity measurement, and ideally, other patient data that each contributes some predictive power.

A systems approach to prevention, meanwhile, emphasizes that intervention in human systems is also complex, and in the case of DFU prevention, likely benefits from components that provide direct patient feedback to sensor-based streams, RPM monitoring of data, and through that human interaction, the repeated emphasis of foot care best practices and other disease management principles. Trials evaluating such strategies are needed, and they may well further clarify the relative value of each layer of defense. However, that search for causal contributions should not preclude a systems-based approach that is likely to deliver immediate improvements to DFU care.

## 11. Conclusions

Diabetes and its associated complications, including DFUs, pose a significant global health burden. DFUs lead to severe consequences such as lower extremity amputations, increased mortality rates, and significant healthcare costs. The current standard of care methods have limitations in early detection and treatment, hindering prevention efforts. Sensor-based RPM programs may help overcome those limitations, encouraging higher adherence to integrative foot care best practices, and complementing them with early warning and patient self-management strategies. Based on our experience in deploying sensor-based preventative RPM programs, we have looked to offer a framework for integrating sensor-based RPM in DFU prevention regimens.

Diabetic foot complications are influenced by various factors, making it important for sensor-based remote patient monitoring (RPM) to collect physiological and behavioral data from multiple domains. Monitoring domains such as pressure, temperature, and activity offer opportunities for RPM services to intervene at different stages of the disease process, drive insights into pathogenesis, and add value to predictive models for DFU. Continuous investment and refinement are necessary for prediction systems to improve accuracy and reduce false positives, which can be burdensome for patients and healthcare teams.

Diabetic foot care delivered through RPM offers an opportunity to become more synergistic with the care plan of patients living with diabetes. Sensor-based monitoring of the diabetic foot should not be viewed in isolation but as a part of a comprehensive disease management approach. The integration of RPM should align with other principles in diabetes care and promote progress toward optimizing glycemic control and reducing cardiovascular risk. Activity monitoring, prescription, and modulation, as well as motivational interviewing, should occupy an important place in a preventative RPM plan informed by sensor-based physiologic data streams. We should seek to reduce the foot health concerns that can serve to create barriers to healthy living so that the metabolic, cardiovascular, and mental health benefits of active lifestyles can be realized by the patients that need them most. In so doing, sensor-based monitoring of the diabetic foot can impact not only foot health but the disease trajectory of diabetes and the healthspan of patients living with this disease.

## Figures and Tables

**Table 1:** Comparison of technology form factor and data measurement domains in existing sensor-based RPM solutions aimed at DFU prevention.

Solution	Podimetrics SmartMat™	Siren Socks	Orpyx SI^®^ Sensory Insole System
Form factor	Mat	Socks	Insole: custom or prefabricated
Data sampling	Once per day	All day	All day
Temperature monitoring	Yes	Yes	Yes
Pressure monitoring	No	No	Yes
Activity monitoring	No	No	Yes

**Table 2:** Remote patient monitoring recommendations based on international guideline risk levels.

Group	Definition	Risk Level	RPM Recommendation
IWGDF Patient Risk Levels			
0	No LOPS *, no PAD *, no FD *	Very low	Not required
1	LOPS + PAD	Low	In the presence of LOPS
2	LOPS + PAD, or LOPS + FD, or PAD + FD	Moderate	With history of previous (re-epithelialized) foot ulcer
3	LOPS or PAD with one or more of: (1) History of foot ulcer; (2) major or minor LEA *; and (3) ESRD *	High	With history of previous (re-epithelialized) foot ulcer
PAVE Patient Risk Levels			
0	No sensory loss, diminished circulation, ulceration, or amputation	Normal	Not required
1	No sensory loss, diminished circulation, ulceration, or amputation, but any of the following: (1) FD; (2) Minor foot infection; (3) Minor diminution of circulation	Low	Not required
2	Sensory loss and may have: (1) Diminished circulation (absent or loss of protective sensation); (2) FD or minor foot infection and diagnosis of diabetes	Medium	With findings suggestive of LOPS, especially if concurrent foot deformity or poor circulation
3	PN + sensory loss and may have diminished circulation, FD, minor foot infection and any of: (1) ulcer or history of prior ulcer; (2) Severe PAD; (3) Charcot + FD; and (4) chronic kidney disease	High	With history of previous (re-epithelialized) foot ulcer

* loss of protective sensation (LOPS), peripheral arterial disease (PAD), foot deformity (FD), lower extremity amputation (LEA), end-stage renal disease (ESRD).

**Table 3:** Clinical history and physical assessment in patients at risk for foot ulceration. Adapted with permission from Ref. [56].

Patient History/Clinical Presentation	Physical Examination
Medical history ○ Arterial macro/micro vessel disease or vascular disease, or both ○ Level of diabetic control Complete surgical history, including any amputations Social history, including substance and tobacco use Nutritional status Wound history ○ Cause and duration ○ Infection history ○ Medical and surgical treatment history Mobility history/Functional status ○ Assistive devices	Vital signs Lower leg assessment ○ Sensory loss (SW 5.07 Monofilament) ○ Vascular assessment ○ Presence or absence of foot deformity or callus, color, or temperature differences Gait assessment Wound assessment ○ Location ○ Measurement ○ Description ○ Presence or absence of infection Environmental factors under foot (dressings, insole modifications with padding, etc.)

## Data Availability

Not applicable.
